# 

**Published:** 1883-06-20

**Authors:** 


### TABLE OF CONTENTS.

ORIGINAL AND SELECTED ARTICLES.

Carbolic Acid in Piles and other Diseases, by J. G. Westmoreland, M. D., Atlanta, 201. On the Treatment of the Commoner Forms of Skin Diseases, by J. Leslie Foley, M. D., London, 203. The Use of Iodine as a Stomachic Sedative, by Thomas T. Gaunt, M.D., New York, 208. Intra-Uterine Injections in the Treatment of Puerperal Scepticæmia, by T. Gaillard Thomas, M. D., 213. Death from the Use of Chloroform, by Hunter McGuire, D.M., of Richmond, 215. Observations on Eclampsia, by H. L. W. Burritt, M.D., 216. How to Cure a Felon, by J. M. Hole, M.D., Salem, Ohio; Treatment of Cerebro-Spinal Meningitis, 217.

ABSTRACTS AND GLEANINGS.

Hot Water in Post-Partum Hemorrhage, 218. Clinical Notes on Opium Addiction, 221. Ulceration of the Os Uteri; Bovine Virus' Disapproved, 224. New Remedy for Syphilis, 225. A Five-Franc Piece Swallowed; Corrosive Sublimate, 226. Treatment of Delirium Tremens; Confidential Communications, 227. Removal of Warts; Eucalyptus, 228. A Foreign Body in the Air-Passages, 229. Diagnosis of Consumption; Fistula Ani; Volta Prize; Quinine Amaurosis; Colic, 230.

SCIENTIFIC ITEMS.

The Afflictions of the Spectroscope, 231. Transit of Venus; A Man; It is Reported; A Swede, 232.

PRACTICAL NOTES AND FORMULÆ.

The Odor of Iodoform; A Little Girl; Aphthous Sore Mouth in Infants, 233. Hop Cordial; Remedy for Baldness; Cracked Nipples; Emmenagogue, 234. To Kill Rats and Mice; Expectorant Mixture in Bronchitis; A Specific for Singultus, 235.

EDITORIALS AND MISCELLANEOUS.

Editorial Notices, 236. Medical Journalism as a Teacher, 237. Book Notices, 238. Receipted; Special Notices, 240.

